# Annotations

**Published:** 1903-07-11

**Authors:** 


### ANNOTATIONS.

### Hospital Finance.

IN the *Times* of the 2nd instant there is a letter signed "J. B." which is written "to press upon mercantile houses the duty of annual systematic giving in proportion to income, and to echo from commercial circles the recent pleading at St. Paul's Cathedral for more assured and more continuous support." It is fair to assume that "J. B." is himself the head of a great mercantile house in London, and that his letter is the outcome of discussions he has had with others similarly situated. If this conclusion is correct the letter marks an important departure, and should lead to the adoption of "J. B.'s" plan that all our commercial houses should establish as a rule of business the writing off to a charity account of a certain percentage of their profits, and that each should annually earmark a proportion of such sums to hospitals. Such a charity account should become more and more general among all well-organised businesses, and should have a tendency to automatically increase as each business venture prospers and extends. "J. B." properly maintains that with the growth of income among firms and individuals, the contribution to hospital funds should certainly progress with prosperity. Last year over 600 millions of income was taxed in the United Kingdom, and 1 per cent. on that amount represents six millions sterling, with a prospect, as the national income is expanding at the rate of six millions a year, that the charity account would increase something like £60,000 annually. These figures should give great satisfaction to hospital managers throughout the country, and the plea of "J. B." should encourage them to enforce economy and so to win the confidence of the business community. "J. B." inclines to the view that as national wealth advances the expenditure on the poor relieved by rates and other forms of relief may diminish. Hospital needs on the other hand must always be on the increase if they are adequately to fulfil the requirements of the community. The increase in hospital expenditure is due to the immense advance in methods of treatment during recent years which renders it possible for cases to be relieved nowadays which a few years ago were considered hopeless. We have pointed out over and over again that the hospital population now comprises not only very poor people, but many of those far above the humblest. No doubt the number of commercial houses who have adopted the practice of applying continuously to charity a fixed percentage of their income is at present comparatively small. "J. B." further points out that limited companies as a rule have ample powers to create pension and other funds for the benefit of their staffs, and it behoves them, as their employées enjoy the benefits of hospitals, to make the hospital fund account part of their annual financial arrangements, like the "Reserve" and "Depreciation" accounts. "J. B.", whose firm appears to have adopted the plan he proposes, states as the result of this experience that each of the commercial undertakings which adopts his recommendation will have the satisfaction of knowing that the value of such a system will certainly be reflected in the general conduct of the business, as well as in its greater profit and stability.

### Special Hospitals and Amalgamation.

KING EDWARD'S Hospital Fund has done much to justify the confidence which the public has wisely placed in its council. The latest important reform which has its origin in the action of King Edward's Fund is the amalgamation of the three orthopædic hospitals under one management. The hospital provision for orthopædic cases would then consist of two establishments, namely one central hospital, and a convalescent home or outpost hospital in a suburb of London. The site selected for the new central hospital is that of the National Orthopædic Hospital, in Great Portland Street, with extensions on an adjacent site generously granted by Lord Howard de Walden. All the beds in the new hospital are to be free, and it will be under the management of a combined committee of the existing hospitals which amalgamate, whilst the medical services will be rendered by a staff representing the combined medical staffs of the amalgamated hospitals. The Distribution Committee of the King's Fund are prepared to recommend a grant of £10,000 towards the cost of the amalgamation of the three orthopædic hospitals, and of the necessary buildings, together with an annual grant of £2,000 per annum for three years. The Royal and National Orthopædic Hospitals have agreed to amalgamate, but the City Orthopædic Hospital has declined to join. Should the City Hospital Committee adhere to this decision, and we can hardly think they will, seeing that their accommodation and buildings are at present amongst the least efficient in London, the contribution of the King's Fund is to be reduced one-fourth, and the managers of the City Hospital must be prepared to find, that the public will withdraw their subscriptions in favour of the well-found, modern, and up-to-date orthopædic hospital which London will then possess. We congratulate the King's Fund and the Royal and National Orthopædic Hospitals upon this important new departure, which is calculated to render substantial service to the public by promoting greater efficiency in the treatment of patients, and greater economy in the cost of administration.

### London Accommodation.

THE visit of the French President brought a large number of people into London, but many were caused inconvenience and fatigue owing to a report, which appeared in the Press, and largely credited, that no sleeping accommodation was available within reasonable distance of the busy part of the metropolis. Consequently visitors tried to accomplish sight-seeing and entertainments in one day which should have occupied considerably more time from a hygienic point of view alone. As a matter of fact, though London is fuller than last year at this time, there is ample hotel accommodation for all; in fact the number of hotels has increased so much of late that it is a matter for conjecture if there are not already more than are needed. It is difficult, therefore, to understand what purpose could be served by spreading these false reports to the disadvantage alike of the country cousin and the trade of London.

